# Supplementary material for: A mix-method investigation on acculturative stress among Pakistani students in China
Source: PLoS One. 2020 Oct 2;15(10):e0240103. doi: 10.1371/journal.pone.0240103 (PMC7531799; doi:10.1371/journal.pone.0240103)
Supplement: Attachment — Submitted filename: Response to Reviewer.docx [file pone.0240103.s003.docx]

**Reviewer #2: Major point**

1. A part of literature review must address previous studying conducted on Pakistani students in China, and what these studies have explored. The same can be used to argue/support the findings in the discussion. However, all this is missing in the current article.

Response 1

Literature review section of the article has been modified and revised based on the previous literature found and suggested by respected reviewer 2. The below mentioned two empirical studies were referred to and cited in the literature review part as well as discussed, their findings in the discussion part of this study where needed. The two previous studies had found the factor like language barriers, new academic and social environment, cultural differences responsible for hindering intercultural adaptation but ultimately students learned effective coping strategies and student’s length of stay in China played a crucial role in overcoming the issues of anxiety and depression in China. Similarly, as this Mix Method Study mainly focuses on Pakistani Students acculturations and aims to see the level of stress due to acculturation among Pakistani students in China, mostly comes up with the similar findings and in concordance with the findings like language acquisition provides new students with confidence that enhance social and academic life of students in a cross-cultural environment.

*1. Noreen, Sehrish, Fan Wei Wei, Mehvish Zareen, and Sameena Malik. 2019. "The Intercultural Adjustment of Pakistani students at Chinese Universities." INTERNATIONAL JOURNAL OF ACADEMIC RESEARCH IN BUSINESS AND SOCIAL SCIENCES 9 (3).
2. Su, Xiaoqing. 2017. "The Intercultural Adaptation of the Pakistani Students at Chinese Universities." Universal Journal of Educational Research 5 (12): 2236-2240.*
